# Catalytic-independent roles of chromatin modifiers in mammalian development and the implications for treating human disease

**DOI:** 10.1042/BST20253098

**Published:** 2025-12-19

**Authors:** Hannah K. Vanyai, Marnie E. Blewitt

**Affiliations:** 1 Walter and Eliza Hall Institute of Medical Research, Melbourne, VIC, 3052, Australia; 2 The University of Melbourne, Melbourne, VIC, 3052, Australia

**Keywords:** catalytic function, chromatin modifiers, epigenetics, mammalian embryogenesis, non-catalytic function

## Abstract

The molecular function of chromatin modifiers is canonically assumed to be directly related to their enzymatic activities and these activities are typically measured when investigating the molecular consequences of manipulation in model systems. However, it is increasingly apparent that chromatin modifiers exhibit various functions beyond purely their enzymatic roles and, surprisingly, this is true across many classes of so-called ‘writers’ and ‘erasers’, including histone acetylases and methylases, and histone deacetylases and demethylases. Some of the most striking examples of the catalytic-independent roles of chromatin modifiers can be demonstrated in mouse models, where catalytic-inactive-encoding mutant alleles, in contrast with null alleles, can prolong survival during embryogenesis or, even more profoundly, allow otherwise embryonic lethal mutants to be born and live into adulthood. A deep understanding of the enzymatic and non-enzymatic roles of chromatin regulators is of clear relevance to understanding how they contribute to normal biology and becomes even more relevant given that many of these factors are also now therapeutic targets in the context of disease. Since therapeutic options have expanded beyond small molecule enzymatic inhibitors to include degraders and interaction blocking modalities, the time is ripe to consider these questions. In this review, we explore the catalytic-independent functions of members of four classes of chromatin modifiers, through the lens of mouse embryogenesis where much of the limited *in vivo* data have been reported to date. In addition, we examine how existing mouse models can be assessed to tease apart enzymatic versus non-enzymatic requirements of chromatin modifiers.

## Introduction

Embryonic development, a transcriptionally dynamic and complex period of mammalian life, requires the careful spatial and temporal expression of suites of genes to drive development of different lineages. In this way, a single, identical set of genetic material, i.e. the genome, can steer the single-cell zygote to divide, and divide again, and again, and later to express genes in patterns specific to differentiating lineages and tissue-specific fates, such as heart, gut and brain.

**Figure 1:**
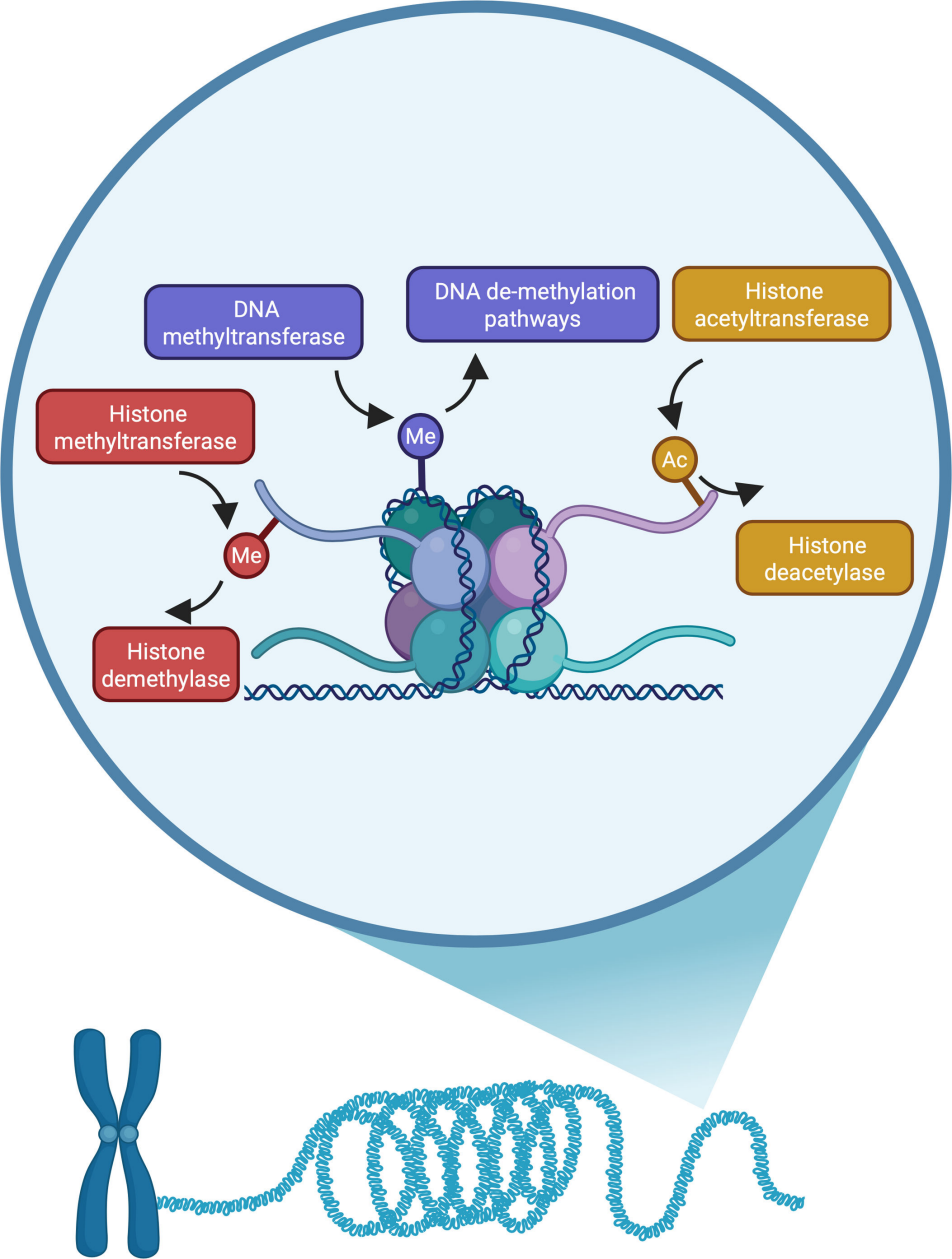
Chromatin enzymes act on nucleosomal histones and DNA. The tails of histone proteins can be decorated with post-translational marks such as acetyl groups (golden boxes) and methyl (red boxes) groups, and DNA can be methylated (purple boxes). The opposing action of chromatin enzymes add or remove these marks in a dynamic manner and this enzymatic function of chromatin enzymes has long been assumed to be their primary, or only, function. Figure created in BioRender.

The mechanisms that enable different sets of genes to be expressed in different cell types heavily involve chromatin modifiers, to package and unpackage genetic material, including genes, into tight, transcriptionally inaccessible regions or more transcriptionally permissible states. The packaging unit is a nucleosome, consisting of a histone octameric protein core wrapped in DNA. Both histone proteins and DNA can be modified by chromatin modifiers through the addition of small chemical groups, which contribute to the packaging of the genetic material (Figure 1). These marks act in concert with other packaging machinery and transcription factors to remodel the chromatin in spatially and temporally controlled processes to enable correct gene expression patterns [1–3].

The histone acetylases and methylases, deacetylases and demethylases, as well as enzymes that place and remove DNA methyl marks, frequently play crucial roles in embryonic development, such that loss of these chromatin modifiers in mouse models results in embryonic lethality. Traditionally, this lethality has been attributed to the loss of the enzymatic function of these proteins as this, surely, is their primary function. However, from recent advances plus a re-examination of mouse models from the last few decades, it is becoming apparent that the function of chromatin modifying enzymes is not enzymatic alone, nor are non-catalytic functions limited to one or a few classes of chromatin modifying enzymes.

There are many classes of chromatin modifiers and we have chosen examples from four of these classes to discuss in this review. Through the enzymatic activity of one chromatin modifier class, histone acetyltransferases such as KAT2A (GCN5) transfer an acetyl mark from the donor acetyl Co-enzyme A to lysines of the histone tails. Another class, the histone deacetylases like HDAC3, catalyse the removal of acetylation. Histone acetylation is associated with gene activity. At the level of DNA, DNA methyltransferases including DNMT3B use the methyl donor S-adenosylmethionine to establish methylation on cytosine bases, which TET1 and other TET dioxygenases can remove through sequential oxidation in the process of active demethylation. The role of DNA methylation in gene expression control is more complex, though DNA methylation at a CpG island promoter of a gene is synonymous with gene silencing.

The broad evidence for the non-catalytic role of epigenetic regulators has recently been the topic of some excellent reviews, largely derived from *in vitro* or non-mammalian studies [4–6]. Here, we consider how the enzymatic versus non-enzymatic roles of a range of chromatin modifiers are being delineated specifically *in vivo* using mouse mutants. Though we will concentrate on models relating to histone acetylation and DNA methylation, *in vivo* evidence of non-enzymatic roles for chromatin modifiers has been suggested for a number of other classes and we point the reader to studies such as those on the histone methyltransferase KMT2A/MLL1 [7,8] and the Polycomb group ubiquitin ligase RING1B [9]. We will focus on embryogenesis, though in the coming years, we expect the work to expand well beyond this period of life. Overwhelmingly, it is apparent that enzymatic-independent functions are also critical for aspects of normal embryogenesis, such that mouse models that carry enzymatic mutant proteins tend to survive longer than mouse models carrying alleles leading to the complete loss of these proteins. These emerging observations suggest that there is much to learn about how these proteins achieve their functions in regulating chromatin state.

### Night and day for embryonic lethality: HDAC3

Perhaps one of the most striking examples of how a catalytic-independent function of a chromatin modifier is critical for embryonic survival is Histone deacetylase 3 (HDAC3). HDAC3 primarily occupies a complex containing either nuclear receptor corepressor (NCOR) or the homologue silencing mediator of retinoic and thyroid receptors (SMRT) [10,11]. Both NCOR and SMRT contain a deacetylase-activating domain (DAD) which interacts with HDAC3, inducing a conformational change that enables the acetylated histone target to enter the catalytic pocket of HDAC3 and be deacetylated [10–13].

**Figure 2:**
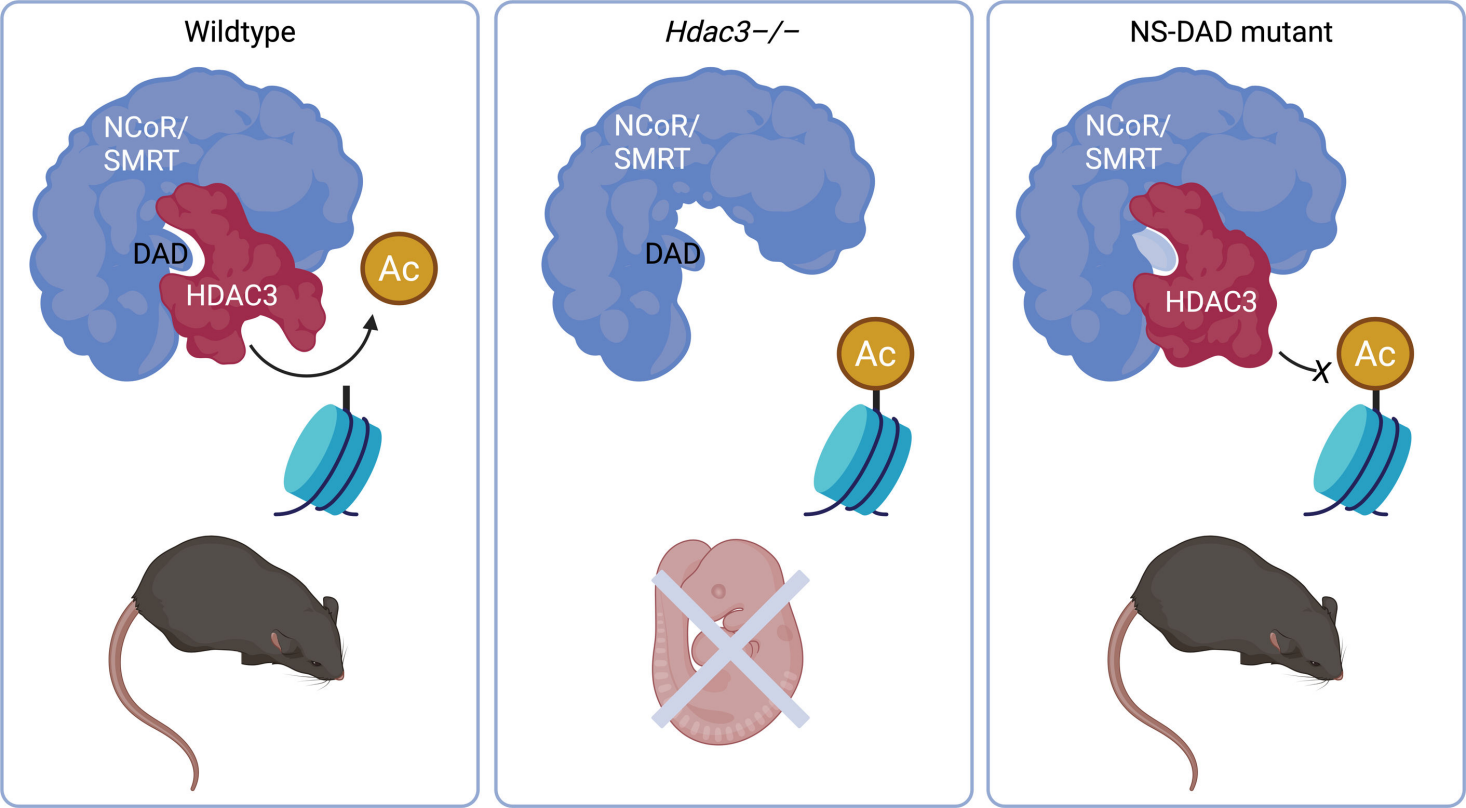
A non-catalytic role of *HDAC3* enables survival into adulthood. HDAC3 requires the deacetylase-activating domain (DAD) of NCoR or SMRT for an open conformation to enable its enzymatic activity (left panel). Embryonic lethality of *Hdac3–/–* animals (middle panel) is not recapitulated in NCoR/SMRT-DAD (NS-DAD) mutant mice (right panel), which renders HDAC3 catalytically inactive and in a closed conformation whilst preserving its binding to the NCoR/SMRT complex. Figure created in BioRender.

Complete loss of HDAC3 from conception leads to embryo death prior to embryonic day (E)9.5 [14,15]. To remove HDAC3 enzymatic activity without, crucially, perturbing the levels of HDAC3 protein, mice carrying double mutation of the NCOR and SMRT DAD domains (NS-DADm) were generated [16]. Remarkably, NS-DADm homozygous mice survive embryogenesis and into adulthood with no change to viability compared with wildtype [16] (Figure 2). Importantly, the authors could demonstrate negligible HDAC3 activity through molecularly isolating HDAC3 and any bound factors from tissues of interest (e.g. adult liver, heart, muscle or whole E12.5 embryo) and performing an *in vitro* deacetylase activity assay. Adults display mild hepatic steatosis, suggesting a requirement for HDAC3 enzymatic function in the adult liver; however, the phenotype is much less severe than for *Hdac3* conditional deletion in the liver [16]. Furthermore, a role for HDAC3 in regulating skeletal muscle fuel metabolism is also highly dependent on the enzymatic function of HDAC3, as NS-DADm and skeletal muscle-specific *Hdac3* mutant mice display parallel impaired muscle function and mass with age [17]. These phenotypes indicate that, though strikingly dispensable for survival through embryonic development, the enzymatic function of HDAC3 is still clearly required for other processes. In a very elegant *in vivo* assay, Sun and colleagues [18] demonstrated that the liver defects caused by depletion of HDAC3 specifically in the liver through injection of AAVS-Cre could be mostly rescued by co-injection of wildtype HDAC3 or a catalytically inactive HDAC3-K25A mutant. However, the specific non-catalytic molecular mechanism was not elucidated.

A study by Lewandowski and colleagues [19] sought to define the non-catalytic function of HDAC3, though, given the difficulties associated with *in vivo* studies, supplemented by *in vitro* exogenous expression studies. They used conditional knockout models to show HDAC3 is required in the second heart field for correct heart development by restraining the TGF-β signalling pathway. Using an explant model of outflow tract cushions from E10.5 embryos, they demonstrated that the unrestrained outgrowth from the cushions in the absence of HDAC3 could be normalised by introduction of either a wildtype HDAC3 or HDAC3 carrying two point variants leading to catalytic inactivity [18,19]. The wildtype or catalytically inactive HDAC3s were both able to recruit PRC2 complex to the *Tgf-β* locus, resulting in histone 3 lysine 27 trimethylation to 100% or 50% (respectively) wildtype levels and equivalently restoring epigenetic silencing of the gene, thereby effecting epigenetic change through recruitment of other chromatin modifying factors.

### Survival of a different class of chromatin modifier mouse mutants

From an entirely different class of chromatin modifiers, namely the DNA methyltransferases, is another striking example where the catalytic function of a protein is dispensable for embryonic development. While all *Dnmt3b ^–/–^* animals perish *in utero* [20,21], Nowialis and colleagues report that animals that are homozygous for a catalytically inactive allele *Dnmt3b^P656V;C657D^* survive [22]. DNMT3B is, along with DNMT3A, a *de novo* DNA methyltransferase, responsible for placing a methyl chemical modification on cytosine bases of DNA, predominantly at CG dinucleotides [20,23]. This function is facilitated in germline development by the closely related but catalytically inactive accessory protein DNMT3L, with which the DNMT3s complex [24,25].

*Dnmt3b^–/–^* embryos perish around E11.5–15.5 due to abnormal heart and neural tube development [20,21]. The survival of *Dnmt3b^P656V;C657D^* homozygous mice suggests that this essential embryonic need for DNMT3B is predominantly related to non-catalytic functions [22]. Nowialis and colleagues suggest that these non-catalytic functions of DNMT3B may be akin to the accessory function of DNMT3L, which is not expressed in somatic cells, and may support the methyltransferase activity of DNMT3A, the other *de novo* methyltransferase. Comparing embryos at E8.5 and 11.5 to understand differences between wildtype, homozygous null and embryos homozygous for the catalytically inactive allele reveals substantially less impact (~5% of total DMRs) on DNA methylation in the catalytically inactive mutant compared with homozygous null animals.

An accessory function has been proposed for DNMT3B previously, both for its catalytically competent isoform as well as for several of its isoforms that lack elements of the C-terminal catalytic domain [26–28]. The accessory activity has been associated with binding to and promoting the methylase activity of either DNMT3A or DNMT3B itself. However, what remains unclear is whether the ability for *Dnmt3b^P656V;C657D^* homozygous animals to survive embryogenesis is because the DNMT3B catalytic function is truly dispensable, or whether the proposed target of the accessory activity and closely related methyltransferase, DNMT3A, is up-regulated, either at the transcript, protein or functional level, in its absence.

### Dual catalytic and non-catalytic requirements during embryonic development

HDAC3 and DNMT3B examples are the most striking demonstrations of improved embryonic survival in catalytic dead versus null models. Next, we will detail an example of more subtle improvements in embryonic development in a member of a different chromatin modifier class.

KAT2A (GCN5) is a histone acetyltransferase which places acetyl groups on histone tails, predominantly at histone 3 lysine (K) 9 as well K14, K18 and K36, as a member of multi-component complexes including STAGA (SPT3-TAF(II)31-GCN5L acetylase) and TFTC (TATA-binding protein-free TAF-containing complex). Homozygous loss of KAT2A leads to mid-gestation embryonic lethality around E9.5 due to aberrant apoptosis leading to loss of mesoderm-derived structures. Null embryos display defects in neural tube closure as well as vascularisation of the yolk sac [29,30]. In contrast, most embryos carrying two copies of a putative catalytic dead allele, *Kat2a^E568A;D609A^*, progress past mid-gestation, though still perish around E12.5-E16.5 [31]. This suggests that there is a delicate requirement for both catalytic and non-catalytic roles for KAT2A in gestation. Indeed, this interplay has been further examined specifically in craniofacial development [32,33]. The comparison of E16.5 foetuses carrying one *Kat2a* null allele deleted in the neural crest lineage with Wnt1-Cre revealed variation in the severity of craniofacial abnormalities depending on whether the second allele produced no protein (homozygous for the null allele) or produced a non-catalytic protein (compound heterozygote with *Kat2a^E568A;D609A^*). Foetuses with no KAT2A protein in neural crest cells displayed severe nasal clefting and almost complete loss of the craniofacial skeleton. In contrast, the presence of a non-catalytic KAT2A protein was sufficient to avoid nasal clefting and to enable formation of the upper and lower jaws, albeit with some remaining abnormalities [32]. The precise nature of this non-catalytic role remains unclear and the coming years will no doubt see a focus on disentangling temporal and spatial requirements for catalytic versus non-catalytic roles of KAT2A and similar.

### Challenging our view of existing mouse models: when a knockout is not just a knockout

Finally, we will explore the interesting case of TET1, responsible for catalysing the removal of DNA methylation marks such as those laid down by DNMT3B, and what existing mouse models may reveal about TET1’s varying roles in development. In the following discussion, all *Tet1* mutant alleles have been described according to the gene structure of the Ensembl Canonical *Tet1* transcript (ENSMUST00000050826.14) for clarity.

**Figure 3:**
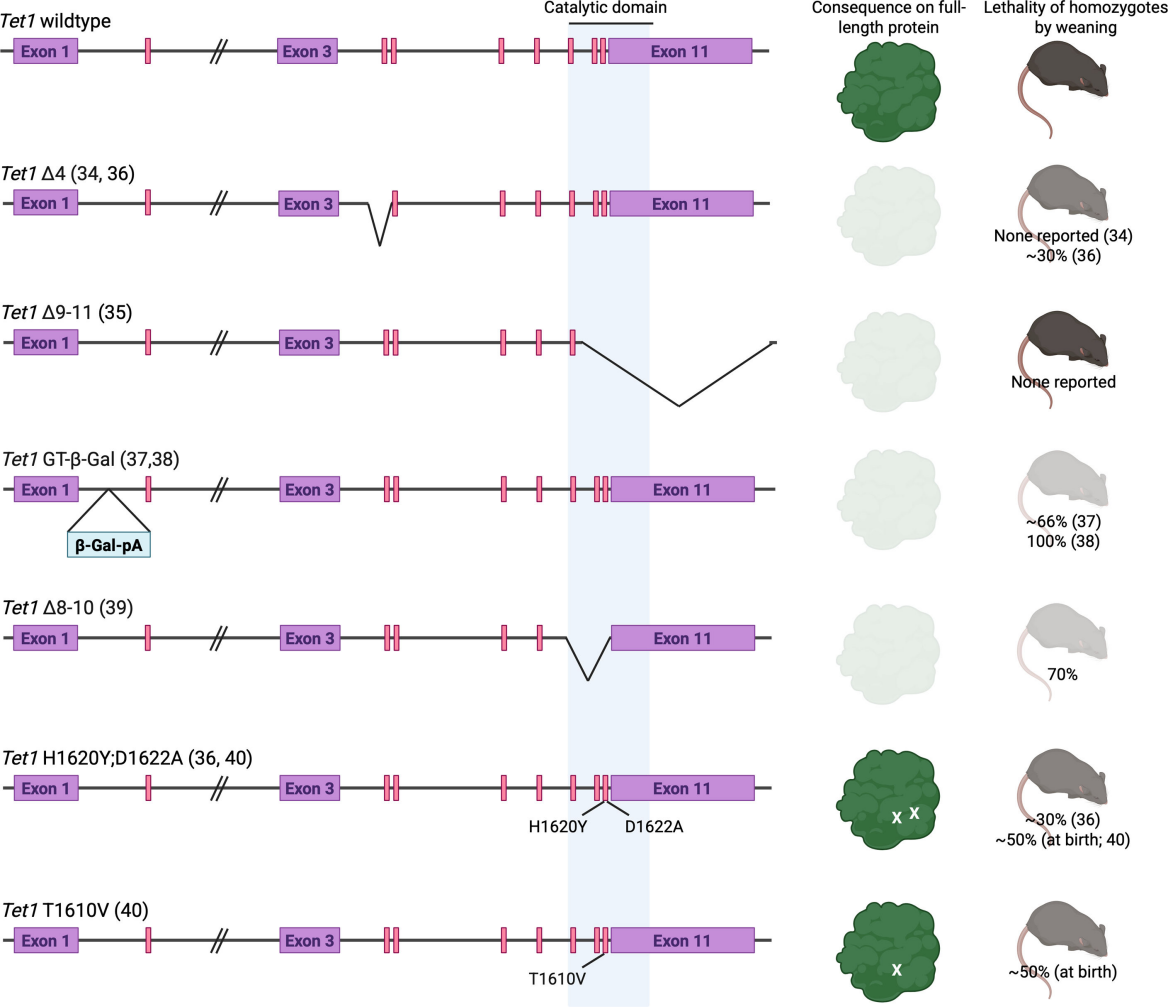
Various mouse *Tet1* mutant alleles and their reported consequences on full-length TET1 protein and homozygous mutant survival. Early studies of *Tet1* null alleles (*Tet1 Δ4* and *Tet1 Δ9–11*) reported no homozygous lethality, whereas further studies reported truncated or putative null alleles resulting in up to 100% neonatal lethality (*Tet1* GT-β-Gal; *Tet1 Δ8–10*). The lower lethality rate of catalytically inactive (*Tet1* H1620Y:D1622A homozygous) or stalled (*Tet1* T1610V homozygous) TET1 mice suggests a possibility of a residual, truncated protein in 3′ *Tet1* null allele-carrying animals and a non-catalytic role for TET1 localising to the N-terminus. *Tet1* mutant alleles described according to the gene structure of the Ensembl Canonical *Tet1* transcript, ENSMUST00000050826.14. Figure created in BioRender.

The earliest mouse models of *Tet1* deficiency, which engineered a deletion of either exon 4 (*Tet1 Δ4*, leading to no protein product [34];) or exons 9–11(*Tet1 Δ9–11*, no protein product detected [35];), resulted in viable homozygous mutant adults and no description of loss prior to weaning, which led to the conclusion that TET1 is dispensable for survival through the embryonic and early postnatal periods. However, closer examination of the *Tet1 Δ4* strain revealed 30% loss of homozygous mutants by weaning [36]. Two further mouse models (*Tet1* gene trap between exons 1 and 2, *Tet1* GT-β-Gal [30,37,38]; deletion of exons 9–12, *Tet1 Δ8–10* [39]) lead to complete embryonic lethality around E9.5 (*Tet1* GT-β-Gal) or 70% embryonic lethality (*Tet1 Δ8–10*) with the remaining 30% surviving to adulthood. Interestingly, Chrysanthou et al. reported a catalytic mutant strain (*Tet1* H1620Y;D1622A) that displayed a milder, smaller size phenotype in homozygotes compared side by side with *Tet1 Δ4* homozygotes, where mild embryonic delay was observed *in utero* [36] (Figure 3). The viability of catalytic mutant *Tet1* lines was further demonstrated by Prasasya et al., who generated both a catalytic mutant strain with the equivalent variants as Chrysanthou et al. as well as an additional strain with stalled, rather than ablated, oxidative activity (*Tet1* T1610V). Both strains demonstrate ~50% homozygous litter loss by birth compared with mid-gestation survival [40].

The discrepancies between these models are intriguing, especially when considering that the catalytic domain of TET1 is encoded by the 3′ exons (~exons 8–11) and that an N-terminal product, capable of binding to chromatin [41], may still be produced in mutants disrupting these exons. However, why homozygosity of the *Tet1 Δ8–10* allele, presumably being not too dissimilar from the reportedly fully viable *Tet1 Δ9–11* mutants, results in 70% embryonic lethality is unclear. Khoueiry and colleagues have neatly discussed the potential impact of genetic backgrounds on the *Tet1* GT-β-Gal alleles, where the complete embryonic lethality of mixed-background *Tet1* GT-β-Gal homozygotes was partially rescued when backcrossed to the C57Bl/6 background for at least three generations [37]. The possibility of a short, potentially functional N-terminal product [41] raises the intriguing possibility that existing mouse mutants of *Tet1* may represent unintended catalytic mutants that can be used to examine the *in vivo* effects of catalytic versus non-catalytic functions of the TET1 protein.

Regarding the non-catalytic mechanisms of TET1, Prasasya et al. demonstrated that their catalytically inactive and stalled mutants protected the sperm genome from hypermethylation at 75% of DMR sites observed in complete *Tet1* knockout sperm [40]. Rather than actively removing DNMT3-dependent methylation, this suggests that the physical occupancy of TET1 at these loci may be needed to prevent access or to recruit further histone modifiers to repel DNMT3. In a similar vein, Khoueiry et al. [37] used an *in vitro* model of extraembryonic trophoblasts and, on a *Tet1*-null background, demonstrated that exogenous TET1 or a TET1 catalytic mutant was equally able to repress expression of a set of TET1-bound target genes. The authors concluded that this may be due to recruitment of JMJD8, a catalytically dead member of the *Jumonji* family of histone lysine demethylases. JMJD8 may act as a repressor whose activity is dominant to the predicted activating role of TET1, through DNA demethylation, at the same site. Other *in vitro* experiments support the theory of a repressor function of TET1 through direct binding with other epigenetic repressors, namely with SIN3A in mouse ESCs [42] and with HDAC1 in cultured mature adipocytes [43]. Further molecular characterisation of TET1 null versus catalytic activity mutant embryos is needed, to explore if this extends *in vivo*.

### Lineage specificity of enzymatic and non-enzymatic roles

So far in the above, we have concentrated on whole-organism effects of null versus catalytic dead alleles in diverse families of chromatin modifiers. The exact molecular mechanisms that underlie non-catalytic roles for chromatin modifiers have been studied somewhat, though either in non-mammalian organisms that lend themselves to external molecular manipulation (e.g. zebrafish, *Drosophila*) or in cell lines. Whilst these studies are certainly informative and have been reviewed recently [4–6], it is becoming apparent that the roles of chromatin modifiers may display exquisite cell type specificity and therefore studying these potential roles in a single cell line, frequently mouse embryonic stem cells, may preclude identifying the full range of roles. For example, catalytically inactive TET2 in mice leads to myeloid disorders but protects against lymphoid disorders observed in full *Tet2* knockout mice [44], suggesting a specific requirement for the non-catalytic roles of TET2 in the lymphoid lineage. Similarly, the de novo methyltransferase DNMT3A may also have lineage-specific requirements for catalytic versus non-catalytic roles. In a *Dnmt3a* knockout mouse model of MYC-induced T-cell lymphomagenesis, a catalytically dead DNMT3A was able to suppress *Dnmt3b* expression to an equivalent level as the wildtype DNMT3A protein, suggesting non-catalytic suppression of *Dnmt3b* [45]. However, expression of the same catalytically dead *Dnmt3a* allele in *Dnmt3a* knockout neural stem cells could not rescue their differentiation defect, which could otherwise be overcome by expressing wildtype *Dnmt3a* [46]. Such exquisite cell-type specificity highlights the need to study catalytic dead mutants versus full protein knockout or depletion in multiple *in vitro* models, to better capture the different molecular mechanisms of chromatin enzymes across different tissues. Ultimately, studying these variants *in vivo*, likely with conditional models, will be the most illuminating.

### Applying developmental insights to human disease

Epigenetic modifiers are frequently dysregulated in cancers and genetic syndromes, including neurodevelopmental disorders. To develop appropriate treatments for these disorders, it is important to understand the relative contributions of the enzymatic versus non-enzymatic dysregulation in these contexts. For example, a disease state that is driven by non-enzymatic functions of a chromatin modifier may not benefit from treatment by inhibition of the enzymatic domain but may from targeted loss of the whole protein. This concept has gained momentum recently, fuelled by the rise of targeted protein degradation approaches (reviewed in [47]).

SMARCA2/4 [48], EZH2 [49] and p300/CBP [50] are three examples of epigenetic regulators for which total protein degradation is a more effective option than small molecule inhibition. For example, the chromatin remodelling enzymes SMARCA2 and SMARCA4 are required for the proliferative capacity of numerous cancer cell lines [47,51,52]. However, small molecule inhibitors developed to recapitulate the anti-proliferative effects of total protein loss, by targeting either the enzymatic region or the bromodomain, have proven substantially less effective [53,54], indicating that functions of SMARCA2/4 beyond the enzymatic are driving proliferation in the cancer setting. In contrast, targeted protein degradation of SMARCA2/4, using the bromodomain-binding small molecule inhibitor to target PROTAC-driven degradation of the whole protein, robustly inhibits proliferation in numerous SMARCA2/4-dependent cancer cell lines [48]. Use of the bromodomain-binding small molecule inhibitor alone failed to inhibit proliferation, indicating that complete ablation of the protein is required for the anti-proliferative effect, suggesting that the total protein degradation approach is hugely promising as a therapeutic avenue for SMARCA2/4-dependent cancers.

Therefore, there are now multiple examples of epigenetic modifiers, critical for cancer survival, that do not show therapeutic potential with small molecule treatments targeting enzymatic or other key domains but recapitulate the promising anti-proliferative effects of knockdown or knockout tumour models when the full protein is degraded through PROTAC, or similar, systems. Conversely, targeting only the enzymatic function where that is the primary disease driver may be of benefit due to fewer off-target effects if the remainder of the protein is retained and may be of particular importance in the case of paediatric cancers in which the cancer may rely on the enzymatic activity of an epigenetic modifier.

A related point to the role of the catalytic activity of chromatin modifiers is the role the chromatin marks imparted by some of these enzymes play in gene regulation. While historically the specific marks themselves were assumed to play a critical role, it has only very recently been possible to formally test the role of chromatin marks in gene regulation using dead Cas9 tethered chromatin enzymes or their non-catalytic mutants [55]. However, there is not yet sufficient detail from *in vivo* models to ask whether these studies align. In general, given embryonic development appears to be more tolerant to loss of catalytic activity than loss of the whole protein, it is tempting to speculate that the catalytic activities evolved to provide more nuanced regulation to exposures we experience later in life.

**Perspectives**

- Understanding the nuanced contribution of catalytic versus non-catalytic functions of chromatin regulators is critical to develop therapeutics that target the relevant function/s of the chromatin modifiers.
- Chromatin modifying enzymes have many roles beyond the catalytic and studies to date indicate that these roles have a high degree of cell type specificity. These non-catalytic roles seem particularly important during mammalian embryonic development.
- A temporal role for enzymatic functions indicates that researchers need to consider both cell type as well as life stage when defining molecular roles of chromatin modifying enzymes. These data should then be incorporated when interpreting the mechanisms by which chromatin enzymes regulate gene expression, and indeed how they may be targeted in the context of disease.

## Abbreviations

DAD: deacetylase-activating domain
HDAC3: histone deacetylase 3
NCOR: nuclear receptor corepressor
SMRT: silencing mediator of retinoic and thyroid receptor

## References

[1] Allis C.D. Jenuwein T 2016 The molecular hallmarks of epigenetic control Nat. Rev. Genet. 17 487 500 10.1038/nrg.2016.59 27346641

[2] Blackledge N.P. Klose R.J 2021 The molecular principles of gene regulation by Polycomb repressive complexes Nat. Rev. Mol. Cell Biol. 22 815 833 10.1038/s41580-021-00398-y 34400841 PMC7612013

[3] Kouzarides T 2007 Chromatin modifications and their function Cell 128 693 705 10.1016/j.cell.2007.02.005 17320507

[4] Aubert Y. Egolf S. Capell B.C 2019 The unexpected noncatalytic roles of histone modifiers in development and disease Trends Genet 35 645 657 10.1016/j.tig.2019.06.004 31301850 PMC6697606

[5] Morgan M.A.J. Shilatifard A 2020 Reevaluating the roles of histone-modifying enzymes and their associated chromatin modifications in transcriptional regulation Nat. Genet. 52 1271 1281 10.1038/s41588-020-00736-4 33257899

[6] Morgan M.A.J. Shilatifard A 2023 Epigenetic moonlighting: Catalytic-independent functions of histone modifiers in regulating transcription Sci. Adv. 9 eadg6593 10.1126/sciadv.adg6593 37083523 PMC10121172

[7] Mishra B.P. Zaffuto K.M. Artinger E.L. Org T. Mikkola H.K.A. Cheng C et al. 2014 The histone methyltransferase activity of MLL1 is dispensable for hematopoiesis and leukemogenesis Cell Rep 7 1239 1247 10.1016/j.celrep.2014.04.015 24813891 PMC4120120

[8] Terranova R. Agherbi H. Boned A. Meresse S. Djabali M 2006 Histone and DNA methylation defects at Hox genes in mice expressing a SET domain-truncated form of Mll Proc. Natl. Acad. Sci. U.S.A. 103 6629 6634 10.1073/pnas.0507425103 16618927 PMC1440589

[9] Illingworth R.S. Moffat M. Mann A.R. Read D. Hunter C.J. Pradeepa M.M. et al. 2015 The E3 ubiquitin ligase activity of RING1B is not essential for early mouse development Genes Dev. 29 1897 1902 10.1101/gad.268151.115 26385961 PMC4579347

[10] Guenther M.G. Barak O. Lazar M.A 2001 The SMRT and N-CoR corepressors are activating cofactors for histone deacetylase 3 Mol. Cell. Biol. 21 6091 6101 10.1128/MCB.21.18.6091-6101.2001 11509652 PMC87326

[11] Li J. Wang J. Wang J. Nawaz Z. Liu J.M. Qin J. et al. 2000 Both corepressor proteins SMRT and N-CoR exist in large protein complexes containing HDAC3 EMBO J. 19 4342 4350 10.1093/emboj/19.16.4342 10944117 PMC302030

[12] Arrar M. decOliveira C.A.F. McCammon J.A 2013 Inactivating mutation in histone deacetylase 3 stabilizes its active conformation Protein Sci. 22 1306 1312 10.1002/pro.2317 23904210 PMC3795489

[13] Watson P.J. Fairall L. Santos G.M. Schwabe J.W.R 2012 Structure of HDAC3 bound to co-repressor and inositol tetraphosphate Nature 481 335 340 10.1038/nature10728 22230954 PMC3272448

[14] Bhaskara S. Chyla B.J. Amann J.M. Knutson S.K. Cortez D. Sun Z.-W. et al. 2008 Deletion of histone deacetylase 3 reveals critical roles in S phase progression and DNA damage control Mol. Cell 30 61 72 10.1016/j.molcel.2008.02.030 18406327 PMC2373760

[15] Montgomery R.L. Potthoff M.J. Haberland M. Qi X. Matsuzaki S. Humphries K.M. et al. 2008 Maintenance of cardiac energy metabolism by histone deacetylase 3 in mice J. Clin. Invest. 118 3588 3597 10.1172/JCI35847 18830415 PMC2556240

[16] You S.H. Lim H.W. Sun Z. Broache M. Won K.J. Lazar M.A 2013 Nuclear receptor co-repressors are required for the histone-deacetylase activity of HDAC3 in vivo Nat. Struct. Mol. Biol. 20 182 187 10.1038/nsmb.2476 23292142 PMC3565028

[17] Song S. Wen Y. Tong H. Loro E. Gong Y. Liu J. et al. 2019 The HDAC3 enzymatic activity regulates skeletal muscle fuel metabolism J. Mol. Cell Biol. 11 133 143 10.1093/jmcb/mjy066 PMC6392100 30428023

[18] Sun Z. Feng D. Fang B. Mullican S.E. You S.-H. Lim H.-W et al. 2013 Deacetylase-independent function of HDAC3 in transcription and metabolism requires nuclear receptor corepressor Mol. Cell 52 769 782 10.1016/j.molcel.2013.10.022 24268577 PMC3877208

[19] Lewandowski S.L. Janardhan H.P. Trivedi C.M 2015 Histone Deacetylase 3 Coordinates Deacetylase-independent Epigenetic Silencing of Transforming Growth Factor-β1 (TGF-β1) to Orchestrate Second Heart Field Development J. Biol. Chem 290 27067 27089 10.1074/jbc.M115.684753 26420484 PMC4646383

[20] Okano M. Xie S. Li E 1998 Cloning and characterization of a family of novel mammalian DNA (cytosine-5) methyltransferases Nat. Genet. 19 219 220 10.1038/890 9662389

[21] Ueda Y. Okano M. Williams C. Chen T. Georgopoulos K. Li E 2006 Roles for Dnmt3b in mammalian development: a mouse model for the ICF syndrome Development 133 1183 1192 10.1242/dev.02293 16501171

[22] Nowialis P. Lopusna K. Opavska J. Haney S.L. Abraham A. Sheng P. et al. 2019 Catalytically inactive Dnmt3b rescues mouse embryonic development by accessory and repressive functions Nat. Commun. 10 4374 4374 10.1038/s41467-019-12355-7 31558711 PMC6763448

[23] Hsieh C.L 1999 In vivo activity of murine de novo methyltransferases, Dnmt3a and Dnmt3b Mol. Cell. Biol. 19 8211 8218 10.1128/MCB.19.12.8211 10567546 PMC84905

[24] Hata K. Okano M. Lei H. Li E 2002 Dnmt3L cooperates with the Dnmt3 family of de novo DNA methyltransferases to establish maternal imprints in mice Development 129 1983 1993 10.1242/dev.129.8.1983 11934864

[25] Suetake I. Shinozaki F. Miyagawa J. Takeshima H. Tajima S 2004 DNMT3L stimulates the DNA methylation activity of Dnmt3a and Dnmt3b through a direct interaction J. Biol. Chem. 279 27816 27823 10.1074/jbc.M400181200 15105426

[26] Duymich C.E. Charlet J. Yang X. Jones P.A. Liang G 2016 DNMT3B isoforms without catalytic activity stimulate gene body methylation as accessory proteins in somatic cells Nat. Commun 7 11453 10.1038/ncomms11453 27121154 PMC4853477

[27] Gordon C.A. Hartono S.R. Chédin F 2013 Inactive DNMT3B splice variants modulate de novo DNA methylation PLoS ONE 8 e69486 10.1371/journal.pone.0069486 23894490 PMC3716610

[28] Zeng Y. Ren R. Kaur G. Hardikar S. Ying Z. Babcock L. et al. 2020 The inactive Dnmt3b3 isoform preferentially enhances Dnmt3b-mediated DNA methylation Genes Dev. 34 1546 1558 10.1101/gad.341925.120 33004415 PMC7608744

[29] Xu W. Edmondson D.G. Evrard Y.A. Wakamiya M. Behringer R.R. Roth S.Y 2000 Loss of Gcn5l2 leads to increased apoptosis and mesodermal defects during mouse development Nat. Genet. 26 229 232 10.1038/79973 11017084

[30] Yamauchi T. Yamauchi J. Kuwata T. Tamura T. Yamashita T. Bae N. et al. 2000 Distinct but overlapping roles of histone acetylase PCAF and of the closely related PCAF-B/GCN5 in mouse embryogenesis Proc. Natl. Acad. Sci. U.S.A. 97 11303 11306 10.1073/pnas.97.21.11303 11027331 PMC17195

[31] Bu P. Evrard Y.A. Lozano G. Dent S.Y.R 2007 Loss of Gcn5 acetyltransferase activity leads to neural tube closure defects and exencephaly in mouse embryos Mol. Cell. Biol. 27 3405 3416 10.1128/MCB.00066-07 17325035 PMC1899977

[32] Pezoa S.A. Artinger K.B. Niswander L.A 2020 GCN5 acetylation is required for craniofacial chondrocyte maturation Dev. Biol. (NY) 464 24 34 10.1016/j.ydbio.2020.05.006 32446700 PMC9119583

[33] Sen R. Pezoa S.A. Carpio Shull L. Hernandez-Lagunas L. Niswander L.A. Artinger K.B 2018 Kat2a and Kat2b Acetyltransferase Activity Regulates Craniofacial Cartilage and Bone Differentiation in Zebrafish and Mice J. Dev. Biol. 6 27 10.3390/jdb6040027 30424580 PMC6315545

[34] Dawlaty M.M. Ganz K. Powell B.E. Hu Y.-C. Markoulaki S. Cheng A.W. et al. 2011 Tet1 is dispensable for maintaining pluripotency and its loss is compatible with embryonic and postnatal development Cell Stem Cell 9 166 175 10.1016/j.stem.2011.07.010 21816367 PMC3154739

[35] Zhang R.R. Cui Q.Y. Murai K. Lim Y.C. Smith Z.D. Jin S et al. 2013 Tet1 regulates adult hippocampal neurogenesis and cognition Cell Stem Cell 13 237 245 10.1016/j.stem.2013.05.006 23770080 PMC4474382

[36] Chrysanthou S. Tang Q. Lee J. Taylor S.J. Zhao Y. Steidl U. et al. 2022 The DNA dioxygenase Tet1 regulates H3K27 modification and embryonic stem cell biology independent of its catalytic activity Nucleic Acids Res. 50 3169 3189 10.1093/nar/gkac089 35150568 PMC8989540

[37] Khoueiry R. Sohni A. Thienpont B. Luo X. Velde J.V. Bartoccetti M. et al. 2017 Lineage-specific functions of TET1 in the postimplantation mouse embryo Nat. Genet. 49 1061 1072 10.1038/ng.3868 28504700 PMC6033328

[38] Yamaguchi S. Hong K. Liu R. Shen L. Inoue A. Diep D. et al. 2012 Tet1 controls meiosis by regulating meiotic gene expression Nature 492 443 447 10.1038/nature11709 23151479 PMC3528851

[39] Kang J. Lienhard M. Pastor W.A. Chawla A. Novotny M. Tsagaratou A. et al. 2015 Simultaneous deletion of the methylcytosine oxidases Tet1 and Tet3 increases transcriptome variability in early embryogenesis Proc. Natl. Acad. Sci. U.S.A. 112 E4236 45 10.1073/pnas.1510510112 26199412 PMC4534209

[40] Prasasya R.D. Caldwell B.A. Liu Z. Wu S. Leu N.A. Fowler J.M et al. 2024 Iterative oxidation by TET1 is required for reprogramming of imprinting control regions and patterning of mouse sperm hypomethylated regions Dev. Cell 59 1010 1027 10.1016/j.devcel.2024.02.012 38569549 PMC11042979

[41] Zhang W. Xia W. Wang Q. Towers A.J. Chen J. Gao R et al. 2016 Isoform Switch of TET1 Regulates DNA Demethylation and Mouse Development Mol. Cell 64 1062 1073 10.1016/j.molcel.2016.10.030 27916660

[42] Stolz P. Mantero A.S. Tvardovskiy A. Ugur E. Wange L.E. Mulholland C.B. et al. 2022 TET1 regulates gene expression and repression of endogenous retroviruses independent of DNA demethylation Nucleic Acids Res. 50 8491 8511 10.1093/nar/gkac642 35904814 PMC9410877

[43] Damal Villivalam S. You D. Kim J. Lim H.W. Xiao H. Zushin P.-J.H. et al. 2020 TET1 is a beige adipocyte-selective epigenetic suppressor of thermogenesis Nat. Commun. 11 4313 4313 10.1038/s41467-020-18054-y 32855402 PMC7453011

[44] Ito K. Lee J. Chrysanthou S. Zhao Y. Josephs K. Sato H et al. 2019 Non-catalytic Roles of Tet2 Are Essential to Regulate Hematopoietic Stem and Progenitor Cell Homeostasis Cell Rep 28 2480 2490 10.1016/j.celrep.2019.07.094 31484061 PMC6750732

[45] Haney S.L. Hlady R.A. Opavska J. Klinkebiel D. Pirruccello S.J. Dutta S. et al. 2015 Methylation-independent repression of Dnmt3b contributes to oncogenic activity of Dnmt3a in mouse MYC-induced T-cell lymphomagenesis Oncogene 34 5436 5446 10.1038/onc.2014.472 25639876 PMC4533871

[46] Wu H. Coskun V. Tao J. Xie W. Ge W. Yoshikawa K. et al. 2010 Dnmt3a-dependent nonpromoter DNA methylation facilitates transcription of neurogenic genes Science 329 444 448 10.1126/science.1190485 20651149 PMC3539760

[47] Webb T. Craigon C. Ciulli A 2022 Targeting epigenetic modulators using PROTAC degraders: Current status and future perspective Bioorg. Med. Chem. Lett 63 128653 10.1016/j.bmcl.2022.128653 35257896

[48] Farnaby W. Koegl M. Roy M.J. Whitworth C. Diers E. Trainor N. et al. 2019 BAF complex vulnerabilities in cancer demonstrated via structure-based PROTAC design Nat. Chem. Biol. 15 672 680 10.1038/s41589-019-0294-6 31178587 PMC6600871

[49] Ma A. Stratikopoulos E. Park K.-S. Wei J. Martin T.C. Yang X. et al. 2020 Discovery of a first-in-class EZH2 selective degrader Nat. Chem. Biol. 16 214 222 10.1038/s41589-019-0421-4 31819273 PMC6982609

[50] Vannam R. Sayilgan J. Ojeda S. Karakyriakou B. Hu E. Kreuzer J et al. 2021 Targeted degradation of the enhancer lysine acetyltransferases CBP and p300 Cell Chem. Biol 28 503 514 10.1016/j.chembiol.2020.12.004 33400925

[51] Hoffman G.R. Rahal R. Buxton F. Xiang K. McAllister G. Frias E. et al. 2014 Functional epigenetics approach identifies BRM/SMARCA2 as a critical synthetic lethal target in BRG1-deficient cancers Proc. Natl. Acad. Sci. U.S.A. 111 3128 3133 10.1073/pnas.1316793111 24520176 PMC3939885

[52] Shi J. Whyte W.A. Zepeda-Mendoza C.J. Milazzo J.P. Shen C. Roe J.-S. et al. 2013 Role of SWI/SNF in acute leukemia maintenance and enhancer-mediated Myc regulation Genes Dev. 27 2648 2662 10.1101/gad.232710.113 24285714 PMC3877755

[53] Gerstenberger B.S. Trzupek J.D. Tallant C. Fedorov O. Filippakopoulos P. Brennan P.E. et al. 2016 Identification of a Chemical Probe for Family VIII Bromodomains through Optimization of a Fragment Hit J. Med. Chem. 59 4800 4811 10.1021/acs.jmedchem.6b00012 27115555 PMC5034155

[54] Papillon J.P.N. Nakajima K. Adair C.D. Hempel J. Jouk A.O. Karki R.G. et al. 2018 Discovery of Orally Active Inhibitors of Brahma Homolog (BRM)/SMARCA2 ATPase Activity for the Treatment of Brahma Related Gene 1 (BRG1)/SMARCA4-Mutant Cancers J. Med. Chem. 61 10155 10172 10.1021/acs.jmedchem.8b01318 30339381

[55] Policarpi C. Munafò M. Tsagkris S. Carlini V. Hackett J.A 2024 Systematic epigenome editing captures the context-dependent instructive function of chromatin modifications Nat. Genet. 56 1168 1180 10.1038/s41588-024-01706-w 38724747 PMC11176084

